# Research on improved YOLOv8n based potato seedling detection in UAV remote sensing images

**DOI:** 10.3389/fpls.2024.1387350

**Published:** 2024-05-01

**Authors:** Lining Wang, Guanping Wang, Sen Yang, Yan Liu, Xiaoping Yang, Bin Feng, Wei Sun, Hongling Li

**Affiliations:** Mechanical and Electrical Engineering College, Gansu Agricultural University, Lanzhou, Gansu, China

**Keywords:** potato seedling detection, UAV remote sensing, YOLOv8n, lightweight, VanillaNet, GSConv, Slim-Neck

## Abstract

**Introduction:**

Accurate detection of potato seedlings is crucial for obtaining information on potato seedlings and ultimately increasing potato yield. This study aims to enhance the detection of potato seedlings in drone-captured images through a novel lightweight model.

**Methods:**

We established a dataset of drone-captured images of potato seedlings and proposed the VBGS-YOLOv8n model, an improved version of YOLOv8n. This model employs a lighter VanillaNet as the backbone network in-stead of the original YOLOv8n model. To address the small target features of potato seedlings, we introduced a weighted bidirectional feature pyramid network to replace the path aggregation network, reducing information loss between network layers, facilitating rapid multi-scale feature fusion, and enhancing detection performance. Additionally, we incorporated GSConv and Slim-neck designs at the Neck section to balance accuracy while reducing model complexity.

**Results:**

The VBGS-YOLOv8n model, with 1,524,943 parameters and 4.2 billion FLOPs, achieves a precision of 97.1%, a mean average precision of 98.4%, and an inference time of 2.0ms. Comparative tests reveal that VBGS-YOLOv8n strikes a balance between detection accuracy, speed, and model efficiency compared to YOLOv8 and other mainstream networks. Specifically, compared to YOLOv8, the model parameters and FLOPs are reduced by 51.7% and 52.8% respectively, while precision and a mean average precision are improved by 1.4% and 0.8% respectively, and the inference time is reduced by 31.0%.

**Discussion:**

Comparative tests with mainstream models, including YOLOv7, YOLOv5, RetinaNet, and QueryDet, demonstrate that VBGS-YOLOv8n outperforms these models in terms of detection accuracy, speed, and efficiency. The research highlights the effectiveness of VBGS-YOLOv8n in the efficient detection of potato seedlings in drone remote sensing images, providing a valuable reference for subsequent identification and deployment on mobile devices.

## 1 Introduction

In recent years, the global cultivation area for potatoes has remained stable at approximately 20 million hectares, with China’s contribution consistently exceeding 25% (Shi and Xu, 2023). This makes potato cultivation vitally important for food security, economic growth, and poverty alleviation, particularly in densely populated developing countries such as China (Lun et al., 2023). A critical phase in the potato growth cycle is the seedling stage, where accurate detection and counting of seedlings are crucial for predicting yields and achieving high-quality production (Shi et al., 2022). However, traditional manual monitoring methods are costly, inefficient, inaccurate, and often lack representativeness, which impedes the timely and effective implementation of replanting strategies (Lu et al., 2023). The advent of drones, characterized by their agility, compact size, and cost-effectiveness, has increasingly attracted the attention of researchers (Saifizi et al., 2019; Li S. et al., 2023). Utilizing drones in conjunction with deep learning for the automatic detection of crop seedlings presents a simple yet effective method that significantly reduces labor costs and facilitates automation.

Drone platforms, through real-time imagery captured by onboard cameras, have found extensive applications in various fields for target detection (Osco et al., 2020). However, detecting targets from a drone’s perspective often involves dealing with complex environmental backgrounds and small, sometimes blurry, targets. Additionally, the hardware limitations of drones can restrict the complexity of deployable models, leading to less than optimal detection outcomes (Wu et al., 2010; Sishodia et al., 2020). Deep learning algorithms for target detection are generally categorized into two main types: single-stage algorithms, such as Centernet, RetinaNet, SSD, and YOLO, which offer good real-time performance but lower accuracy, particularly in detecting small targets; and two-stage algorithms, like R-CNN, Fast R-CNN, and Faster R-CNN, which provide higher accuracy but at the cost of speed, making them unsuitable for rapid crop information acquisition by drones. The YOLO series, known for its superior performance, has been extensively applied in detection tasks across various domains (Liu et al., 2018; Liang et al., 2022). A current research challenge, and the focus of this study, is leveraging YOLO for accurate and efficient crop seedling detection from a drone’s perspective while maintaining a manageable model size.

The YOLO series models have been broadly applied to drone image datasets. For instance, research by Jianqing Zhao et al. (Zhao et al., 2021) introduced an enhanced YOLOv5 model with an added micro-scale detection layer for wheat ear detection in drone images, achieving a 94.1% accuracy rate, a 10.8% improvement over the standard YOLOv5. However, this method is complex and time-consuming, and the limited memory and processing power available on drones make efficient crop detection challenging. Wang et al. (Wang F et al., 2023) addressed the characteristics of small targets in drone images by embedding a small target detection structure (STC) in the Neck of YOLOv8, capturing comprehensive global and contextual information and incorporating a global attention module (GAM), which significantly improved performance but also increased the parameter count. Li et al. (Li Y. et al., 2023) introduced the concept of Bi-PAN-FPN in YOLOv8 to enhance feature fusion across different scales and utilized the GhostblockV2 structure, achieving an accuracy improvement but falling short compared to other models. Addressing the challenges of insufficient drone computing power and the issue of small targets in drone imagery, Shijie Li (Li, 2023) proposed modifications to the YOLOv5 model, reducing the model’s parameter count from 7.5M to 4.2M, albeit with a 1.7% decrease in detection accuracy. To address the balance between detection accuracy and model size, scholars have conducted relevant research, proposing the use of lightweight convolutional approaches aimed at reducing computational load during the convolution process. For example, Liu et al. (Liu et al., 2022) proposed an improved YOLOv4 model based on MobileNetv2 as the backbone network for orange fruit recognition in orchards, which reduced the model size by 197.5 M and achieved an average recognition accuracy of 97.24%, though the detection time was only reduced by 11.39ms. Rihong Zhang et al (Zhang et al., 2023). introduced a YOLOV4 pineapple seedling heart detection model incorporating a lightweight attention mechanism module CBAM, which reduced the total parameter count by 70% and achieved a recognition accuracy of 95.5%, but the improvement in detection speed was not significant.

While previous methods have shown effectiveness in detecting and counting crops in the field, the unique challenges posed by potato seedlings in UAV imagery—such as their dense distribution, significant overlap, small size, and the complexity of their background, result in a higher likelihood of both false positives and missed detections. These issues compromise the precision of potato seedling detection. Furthermore, the constraints imposed by UAV hardware platforms complicate the task of balancing detection accuracy, speed, and the efficient use of hardware resources. Notably, there is a scarcity of detection methods that are both efficient and specifically tailored to potato seedlings. To address these challenges, this paper introduces a novel lightweight algorithm, VBGS-YOLOv8n. By employing VanillaNet, a network characterized by its simplicity and reduced number of layers, as the backbone network in place of the original YOLOv8n model, we significantly decrease the model’s computational complexity. We enhance the model’s feature fusion capabilities by substituting the PANet path aggregation network with a bidirectional feature pyramid network (BiFPN). Additionally, integrating GSconv convolution within the YOLOv8n’s neck and replacing all C2F networks with the VoV-GCSP module further boosts the model’s performance. This innovative approach facilitates the efficient detection of potato seedlings in UAV remote sensing images, representing a significant advancement in the field.

## 2 Materials and methods

### 2.1 Potato seedling image acquisition

**Figure 1:**
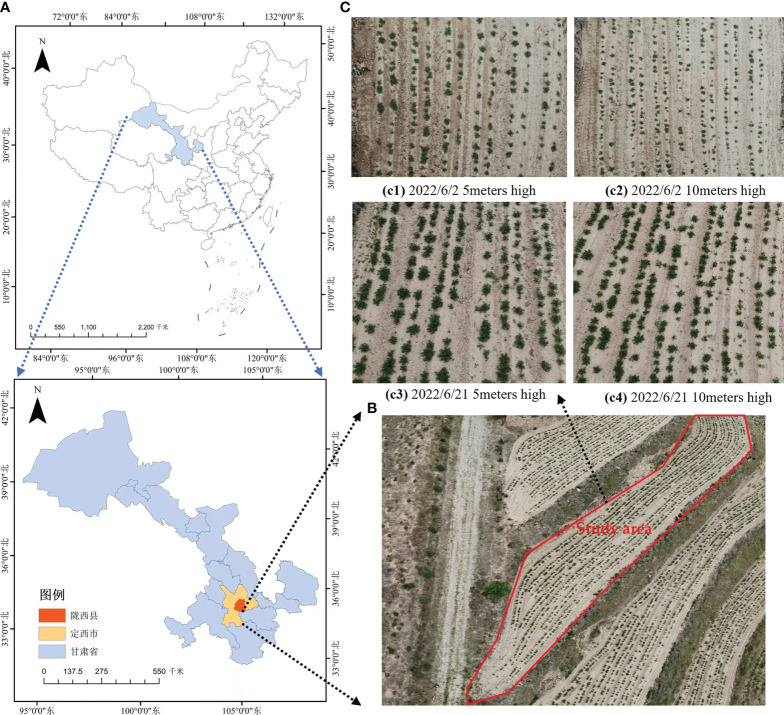
Overview of experimental area and captured images. **(A)** The geographical location of Longxi County, Ding xi City; **(B)** Location of the study area; **(C)** Images of potato seedlings at different heights and growth stages of UAVs.

Potato seedling drone images were collected at Xinghuaping Village, Tonganyi Town, Longxi County, Dingxi City, Gansu Province. The images were captured using a quadcopter drone (DJI Phantom 4 Advanced) and DJI GS Pro. The drone’s RGB camera captured images vertically from above with a shutter speed of 2 seconds. To prevent image blurring, a hover-and-capture method was employed at each waypoint. The front and side overlaps were set at 80% and 70% respectively. The images had a resolution of 4056×3040 pixels and were saved in JPG format. The image collection took place in mid-May and mid-June 2022, between 10:00-12:00. To enhance the model’s ability to generalize for potato seedling detection in various environments, images were collected at drone heights of 5 meters and 10 meters. A total of 409 original images were collected, as shown in **Figure 1**, covering different heights, growth stages and plots.

### 2.2 Dataset construction

**Figure 2:**
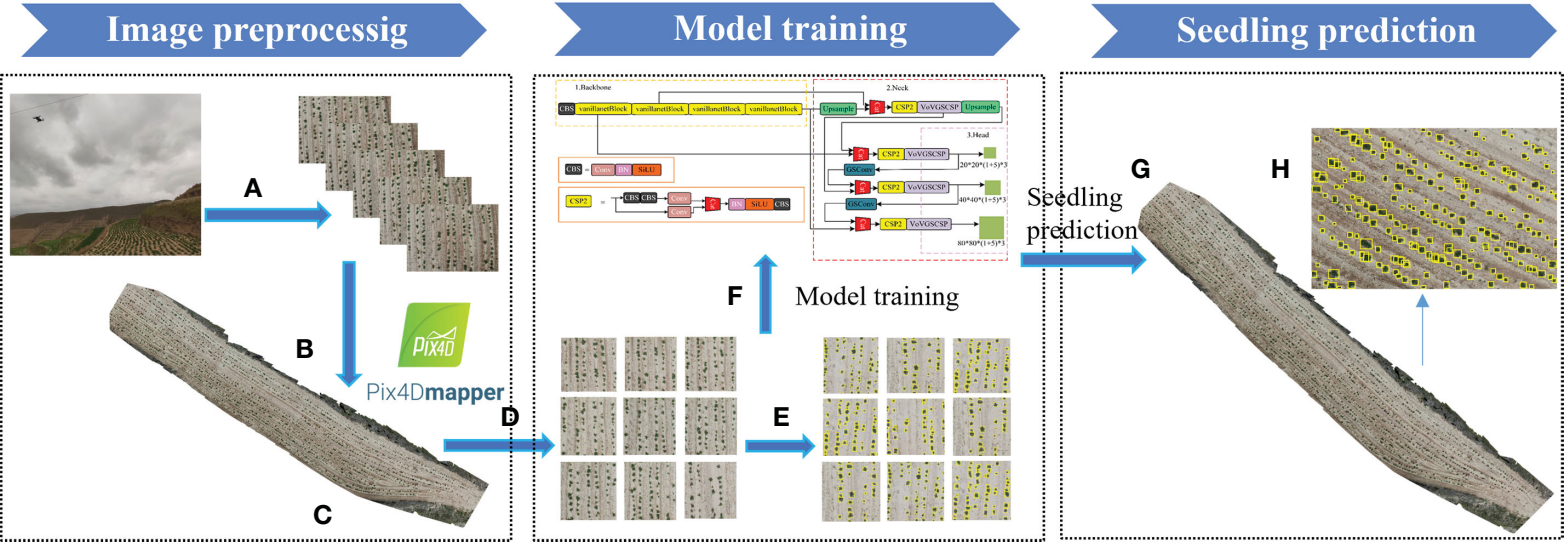
Workflow for image preprocessing and model prediction. **(A)** images taken by UAVs; **(B)** Stitching the images taken by the UAV using Pix4d software; **(C)** Orthophoto generated; **(D)** The large image is cropped into a small image (608 × 608 pixels) for model input; **(E)** annotated image; **(F)** model training; **(G)** The result image of the model prediction output; **(H)** A magnified view of the output image.

**Figure 3:**
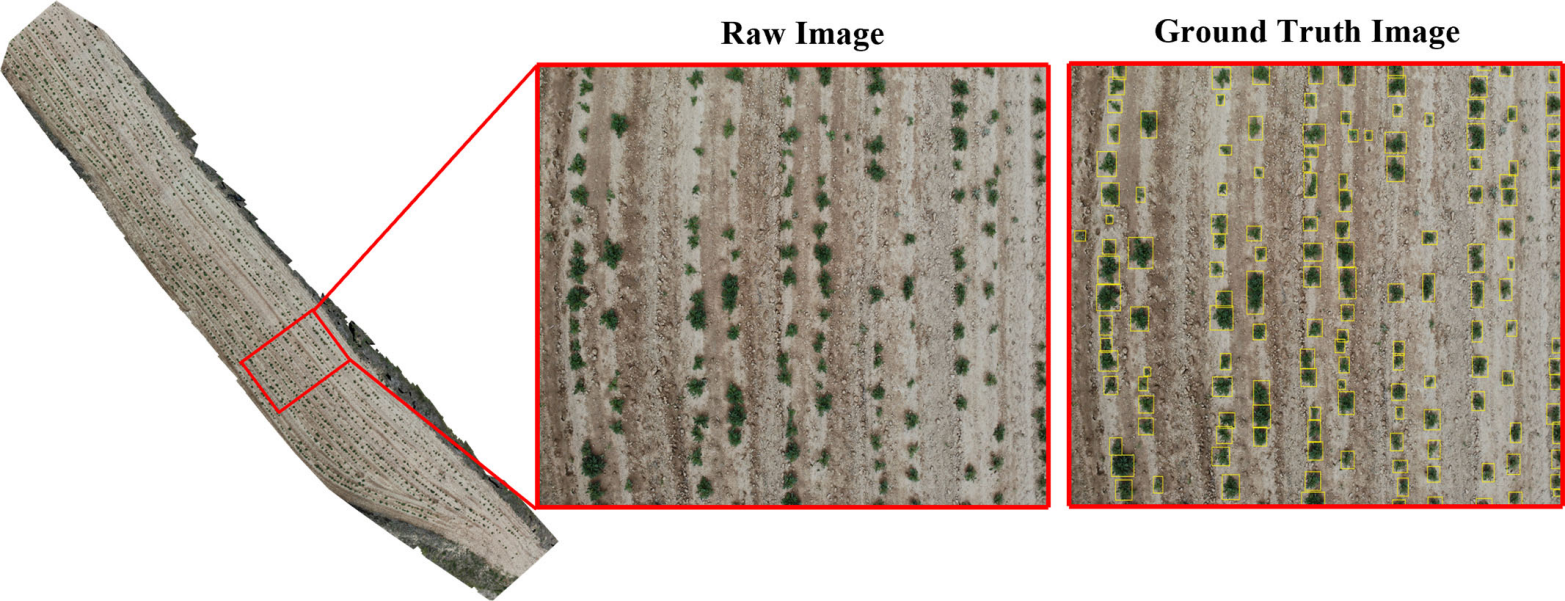
An example of a labeled image used for model training.

The process of potato seedling RGB image detection using the enhanced VBGS-YOLOv8n model is illustrated in **Figure 2**. In this study, Pix4Dmapper software was utilized for rapid stitching and inspection of drone images in the experimental area. During the stitching process, location information was obtained using the GPS system of the drone platform at the time of image capture. Pix4Dmapper then matched approximately 30,000 tie points per original image based on the flight’s POS (Position and Orientation System) data. Subsequently, automatic aerial triangulation technology was employed to calculate the true position data and stitching numbers of the images, leading to the creation of a point cloud model. Following this, the positions and stitching parameters of the original images were automatically optimized and calibrated to generate a Digital Orthophoto Map (DOM) depicting the entire experimental plot (**Figure 2B**). The process resulted in orthophoto images at heights of 5 meters and 10 meters (**Figure 2C**) for two distinct periods. These orthophoto images were then cropped to obtain the dataset images required for model training and prediction (**Figure 2D**). A total of 3089 cropped images were obtained, each with a pixel size of 800×800. To ensure model detection accuracy, 2195 images were selected after screening out unsuitable ones to form the dataset for this study. Manual annotation of the dataset using the LabelImg annotation tool was performed (**Figure 2E**). Subsequently, the improved model (**Figure 2F**) was trained, and the best model after training was used to detect images in the experimental plots (**Figure 2G**), yielding the detection results (**Figure 2H**). During annotation, objects were labeled with bounding boxes that best fit them and assigned the label “seedling,” resulting in the generation of XML files in VOC format. Refer to **Figure 3** for annotated illustrations. Subsequently, the XML files were converted to TXT files required by YOLO using a script. The dataset images and their corresponding TXT files were randomly divided in an 8:1:1 ratio into training set (1754 images), validation set (220 images), and test set (220 images) to adhere to the standard coco format, completing the dataset construction.

### 2.3 Original YOLOv8n

As a one-stage object detection algorithm, YOLOv8 introduces a more lightweight network structure compared to its predecessors, maintaining high accuracy while achieving faster inference speeds. Moreover, YOLOv8 incorporates advanced training methods and techniques, leading to shorter training times and quicker convergence rates. In this study, to balance high detection accuracy with minimal storage usage and enhanced recognition speed for future deployment on mobile devices, the research opts for the YOLOv8n detection model known for its low complexity and lightweight design.

**Figure 4:**
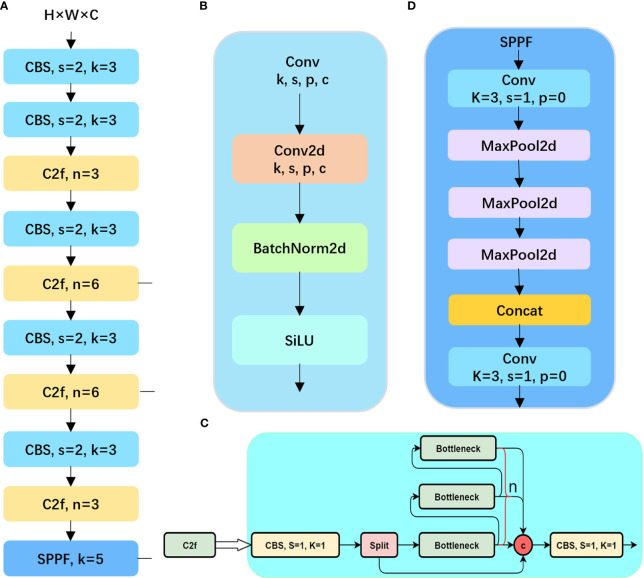
The backbone structure of the yolov8 model and the diagram of each module. **(A)** the overall structure of the backbone; **(B)** the structure of the CBS module; **(C)** the C2f module; **(D)** and the SPPF module.

The YOLOv8n network architecture comprises three main components: the input layer (Input), the backbone network (Backbone), the neck network (Neck), and the detection head (Head). The input layer preprocesses image inputs for the model, while the backbone network, based on CSPDarkNet-53 and utilizing the C2f module, extracts features from input images to generate multi-scale feature maps. The backbone structure is shown in **Figures 4A, B** is a CBS structure diagram. The C2f module in YOLOv8 provides feature fu-sion functionality, which can enhance the performance of object detection, as illustrated in **Figure 4C**. The convolution utilizes CBS, comprising three components: a 2D convolution, 2D BatchNorm, and SiLU activation function. The SiLU activation is computed by multiplying its input with the sigmoid function, i.e., xσ (x). In the case of SPPF, a CBS convolutional layer is followed by three consecutive Maxpooling operations. The feature map without Maxpooling and the feature map obtained after each subsequent Maxpooling operation are concatenated to achieve feature fusion. The structure is shown in **Figure 4D**. The Neck layer adopts the PANet structure, merging feature maps from various scales to capture more global and semantically rich features, thereby enhancing object detection accuracy and recall. The Detect module employs a Decoupled Head, separating regression and prediction branches to predict features across three dimensions, providing class and positional information for the network’s predictions.

In the YOLOv8 model, the loss function plays a crucial role in training the network to accurately predict object bounding boxes and class probabilities. The loss function used in YOLOv8 is a combination of localization loss, confidence loss, and classification loss. The localization loss in YOLOv8 is typically calculated using metrics like Mean Squared Error (MSE) or Smooth L1 Loss. It penalizes the model for inaccuracies in predicting the bounding box coordinates (center coordinates and width/height) compared to the ground truth bounding box. By minimizing the localization loss, the model learns to accurately predict the spatial location and size of objects in the image, improving the precision of object localization. Next, YOLOv8 utilizes binary cross-entropy loss to compute the target confidence loss, assessing the model’s confidence accuracy by comparing predicted target probabilities with ground truth labels. Optimizing the confidence loss enables the model to distinguish objects from the background, enhancing its object detection capabilities. Additionally, the classification loss evaluates the model’s category classification accuracy using binary cross-entropy loss. The calculation formula for classification loss is shown in Equation (1). About Regression Loss, YOLOv8 introduces a Distance-based Focal Loss (DFL) to complement Anchor-Free methods, focusing on optimizing probabilities for the nearest left and right positions to the label y, facilitating quicker convergence on target positions and neighboring regions’ distributions. DFL is calculated as shown in Equation 2.


(1) $$Loss_{cls}=-\sum_{c=1}^{M}y_{o,c}log(p_{o},c)$$

where $y_{o,c}$ is an indicator. 1 if sample *o* belongs to category *c*, and 0 vice versa. $p_{o}$ is the probability that the model predicts that sample o belongs to category *c*.


(2) $$\text{DFL(}S_{i},S_{i+1})=-\left(\left(y_{i+1}-y)\log(S_{i})+(y-y_{i})\log(S_{i+1}\right)\right)$$

The detailed conversion process of transforming labels into DFL format is as follows: y = distance from the center to a specific edge/current downsampling ratio.

The Bounding Box Loss calculates the sum of squared differences between the predicted and actual coordinates, as depicted in Equation 3.


(3) $$Loss_{bbox}\;=\sum_{i=1}^{N}\;{\left(x_{i}\;-{\overset{\wedge }{x}}_{i}\right)}^{2}$$

where $x_{i}$ represents the coordinates of the true bounding box, and ${\hat{x}}_{i}$ represents the coordinates of the predicted bounding box. The loss function is used as the optimization goal to guide the model to reduce the gap between the prediction box and the real box during the training process.

### 2.4 Improvement of the YOLOv8n model

#### 2.4.1 VBGS-YOLOv8n model structure

The YOLOv8n object detection model has been widely applied in the agricultural field due to its excellent recognition accuracy and speed (Sapkota et al., 2023; Wang G et al., 2023). However, the detection of potato seedlings poses some challenges as it involves small target detection tasks. For instance, when deploying the detection task to mobile devices, it is necessary to consider the lightweight nature of the network structure and the reduction of device power consumption. Additionally, due to the small size and overlapping nature of potato seedlings captured by UAVs, there is a risk of missed detections and low accuracy in small target detection. Therefore, this paper proposes a VBGS-YOLOv8n deep learning algorithm based on the YOLOv8n, aiming to achieve higher detection accuracy and a more lightweight model design to better recognize potato seedlings. First, lightweight improvements were made to the backbone, followed by the introduction of the weighted bidirectional feature pyramid network (BiFPN) at the Neck layer, along with the GSConv network, replacing the c2f module with VoV-GSCSP.

**Figure 5:**
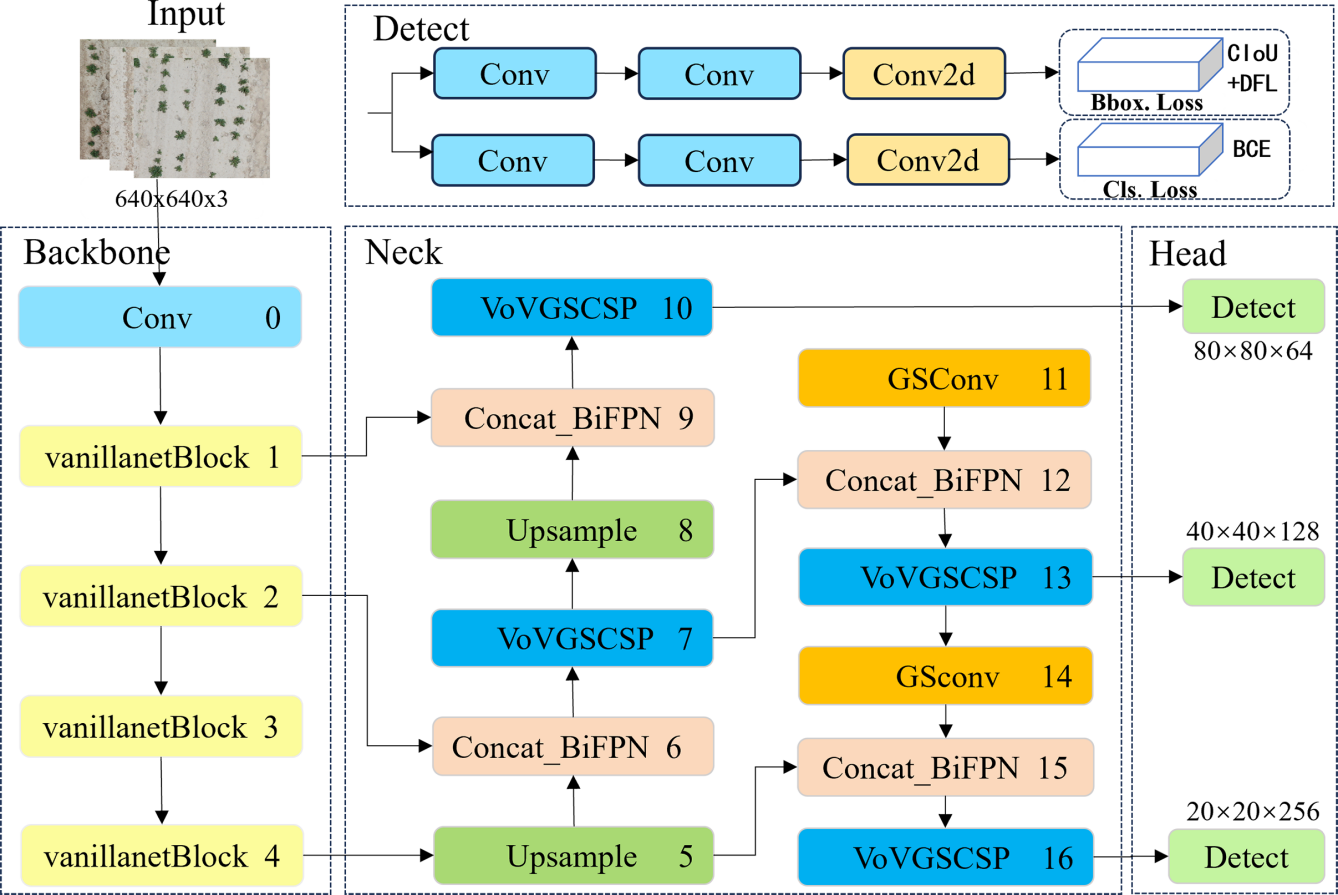
The network architecture diagram of the improved VBGS-YOLOv8n.

The structural design of the proposed VBGS-YOLOv8n model, as depicted in **Figure 5**, involves replacing the CSPDarkNet network of the original YOLOv8 with the lightweight VanillaNet algorithm. The backbone network comprises the initial 4 layers of VBGS-YOLOv8n, starting with a 640*640 RGB image input. With a stride of 4 and double downsampling, spatial feature extraction and data normalization convolution processing are applied, resulting in a halved image resolution. This processed image is then fed into the VanillaNet backbone network. Within the backbone network, stages 1, 2, and 3 utilize max-pooling layers with a stride of 2 to reduce spatial dimensions while retaining crucial feature information, doubling the channel count at each layer. Stage 3, representing the third layer of the network, undergoes an 8x downsampling to yield an image with 512 channels. Stage 4 maintains the channel count without increase, following an average pooling layer. The final layer consists of a fully connected layer for classification output with a stride of 1. Each layer in the VanillaNet backbone network employs 1x1 convolution kernels to preserve feature map details efficiently. The input features are downsampled to appropriate sizes, resulting in image resolutions of 160*160, 160*160, and 80*80 at Layer 1, Layer 2, and Layer 4, respectively.

The 1st, 3rd, and 4th layers serve as inputs to the neck structure. In contrast to the PANet bidirectional pathway network used in the original YOLOv8n network’s neck structure, the VBGS-YOLOv8n model integrates a BiFPN with adjustable weights in each concat module of the neck network for feature extraction. The BiFPN facilitates more efficient multi-scale feature fusion. Furthermore, the c2f modules at each layer are replaced with the cross-level subnetwork VoV-GSCSP module. Additionally, GSConv convolution is applied at the 11th and 14th layers of VBGS-YOLOv8n, aiming to reduce computational costs and maintain inter-channel connections effectively. Through a process of layer-wise upsampling and feature concatenation, diverse scale feature information is fused. By the 16th layer of the model, the number of output channels in the image is increased to 1024.Subsequently, the three output branches from the neck are directed to the detection head for loss computation or result inference. YOLOv8 introduces a decoupled head, replacing the coupled head of previous YOLO models. This decoupled head separates the regression and prediction branches, utilizing the integral form proposed in the distribution focal loss strategy for the regression branch. The decoupled head exhibits faster convergence and improved performance. In VBGS-YOLOv8n, the head network generates images of sizes 80×80, 40×40, and 20×20 for potato seedling detection.

#### 2.4.2 Lightweight backbone network

VanillaNet, a lightweight neural network architecture that emphasizes simplicity, was introduced by Huawei’s Noah’s Ark Lab (Chen et al., 2023). By avoiding complexities like excessive depth, shortcuts, and self-attention mechanisms, VanillaNet achieves a balance of simplicity and performance. Overcoming the inherent complexity of traditional deep networks, VanillaNet emerges as an optimal choice for environments with limited resources. Its streamlined architecture not only enhances comprehension but also provides an effective solution for efficiently deploying potato seedling detection in drone-based remote sensing applications.

**Figure 6:**
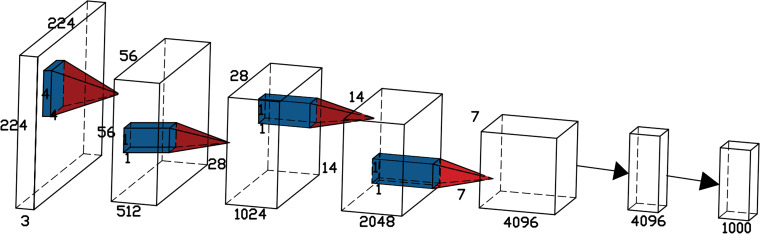
The architecture of the VanillaNet-6 consisting of only 6 convolutional layers.

VanillaNet is characterized by the absence of convolution layers and branches in its network structure, as depicted in **Figure 6**. The network comprises a backbone, main body, fully connected layers, and 5 activation functions. The design principle follows a gradual reduction in resolution and an increase in channel numbers, without incorporating shortcuts, attention mechanisms, or other computations.

For the backbone, a 4×4×3×C convolution layer is utilized with a stride of 4, following common configurations from [18,31,32], to transform 3-channel images into features with C channels. In stages 1, 2, and 3, max-pooling layers with a stride of 2 are used to decrease size and feature maps while doubling the channel count. Stage 4 maintains the channel count unchanged by employing average pooling. The final fully connected layer is dedicated to producing classification outcomes. Each convolution layer employs a 1×1 kernel to retain feature map details while minimizing computational costs. Batch Normalization (BN) is applied after each layer to streamline the training process and enhance the simplicity of the architecture. This approach achieves an optimal trade-off between speed and accuracy, showcasing the excellence of VanillaNet.

While VanillaNet’s simple structure is easy to implement, its limited nonlinearity hinders network performance enhancement. To tackle this challenge, the authors introduce a deep training strategy and incorporate a series-inspired activation function to boost the network’s nonlinear expressive capacity.

The deep training strategy involves splitting the network into two convolution layers, increasing the network depth only during training, and merging them during inference. This approach reduces network computation and complexity. The split convolution layers will utilize the following Equation 4 activation function:


(4) $$A^{'}(x)=(1-\lambda )A(x)+\lambda x$$

When training converges, the two convolutional layers without non-linear activation are merged into one layer, achieving the effect of deep training and shallow inference.

(1) Activation Function Inspired by Series: Concurrently stacking activation functions can significantly enhance the non-linearity of the activation function. Representing the single activation function of the input in the neural network as A(x) Equation 5:


(5) $$A_{s}\left(x\right)=\sum_{i=1}^{n}a_{i}A\left(x+b_{i}\right)$$

In the equation, n represents the number of stacked activation functions, while $a_{i}$ , $b_{i}$ are the scale and bias of each activation to avoid simple accumulation. To further enrich the sequence, given an input feature $x\in R^{H\times W\times C}$ where H , W and C are its width, height, and number of channels, the activation function is formulated as Equation 6:


(6) $$A_{s}(x_{h},w,c)=\sum_{i,j\in \{-n,n\}}a_{i,j,c}A(x_{i+h,j+w,c}+b_{c})$$

From the equation, it can be found that when n=0 , the proposed method can be regarded as a general extension of existing activation functions.

The computational complexity expression of the proposed activation function O(CONV) compared to its corresponding convolutional layer is shown in Equation 7).


(7) $$\frac{O(\text{CONV})}{O(\text{SA})}=\frac{H\times W\times C_{\text{in}}\times C_{\text{out}}\times K^{2}}{H\times W\times C_{\text{in}}\times n^{2}}=\frac{C_{out}\times k^{2}}{n^{2}}$$

In the equation, $C_{\text{in}}$ represents the input channels, $C_{out}$ represents the output channels, and k represents the kernel size. Taking the fourth stage of VanillaNet-B as an example, where $C_{out}$ = 2048, k = 1, n = 7, the ratio is only 84, indicating that the computational cost of this activation function is much lower than that of a convolutional layer. Therefore, the use of these two non-linear solutions can significantly improve the detection accuracy of VanillaNet.

#### 2.4.3 BiFPN feature fusion

**Figure 7:**
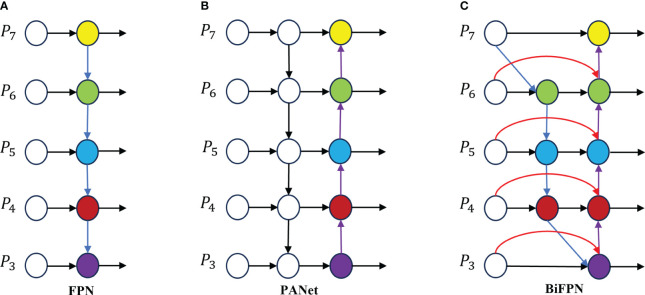
Feature network design **(A)** FPN network; **(B)** the principle of PANet; **(C)** is BiFPN schematic.

Feature fusion is a critical aspect in object detection, aiding in the extraction of information from various scales to enhance detection accuracy. The traditional Feature Pyramid Network (FPN) structure serves as a method for feature fusion, integrating a top-down pathway to merge multi-scale features from levels 3 to 7 (P3 to P7), as depicted in **Figure 7**. Expanding on FPN, the YOLOv8 feature extraction network incorporates PANet (**Figure 7B**), which introduces an additional bottom-up pathway aggregation network to FPN (**Figure 7A**). However, these fusion methods can lead to information loss or feature redundancy (Wang Y et al., 2023). This study introduces an efficient BiFPN (**Figure 7C**) structure that leverages effective bidirectional cross-scale connections and weighted feature fusion. By adjusting feature map scales through upsampling and downsampling operations, different scale features are fused to preserve finer details, thereby improving small object detection accuracy.

BiFPN (Tan et al., 2020) is a network structure that efficiently incorporates repeated bidirectional cross-scale connections and weighted feature fusion. In comparison to PANet, BiFPN eliminates nodes with single input edges that do not merge different features, making it lighter and faster in inference speed with fewer parameters. Additionally, an extra edge is introduced between the original input and output nodes at the same layer to enhance the fusion of additional image features. By leveraging bidirectional repeated connections for information fusion, feature details are preserved, enhancing accuracy in small object detection. BiFPN utilizes a weighted feature fusion mechanism that differentiates and merges various input features through learning, adapting to different resolutions, and addressing feature loss issues caused by simple overlaying of feature maps. It serves as a straightforward and efficient feature fusion approach. BiFPN adopts the Fast Normalized Fusion method, akin to Softmax, mapping each input value to the range [0, 1], thereby improving training speed and efficiency, enhancing data consistency and comparability for better analysis and decision-making, as depicted in Equation (8).


(8) $$O=\sum_{i}\frac{w_{i}*I_{i}}{\varepsilon +\sum_{j}w_{j}}$$

In the equation, $I_{i}$ represents the input features, $w_{i}$ and $w_{j}$ denote the weights obtained during network training, ε = 0.0001.

#### 2.4.4 GSConv network and Slim-Neck design paradigm

In order to achieve real-time object detection on mobile devices, reducing model complexity, enhancing detection speed, and maintaining high accuracy are essential for the task of potato seedling image detection captured by drones. GSConv+Slim-Neck is a lightweight network proposed for a vehicle-mounted edge autonomous driving computing platform (Li et al., 2022). This network design aims to facilitate efficient object detection to meet real-time application requirements. GSConv strikes a balance between model accuracy and speed, enabling model lightweighting while preserving accuracy. Introducing GSConv provides a design paradigm called Slim-Neck, which utilizes a one-time aggregation method to create the cross-level subnetwork (GSCSP) module VoV-GSCSP. This module reduces computational and network structural complexity, thereby enhancing detection accuracy. Hence, this paper adopts this network to reduce model complexity, enhance detection speed, and maintain high accuracy for mobile deployment, offering an effective solution.

**Figure 8:**
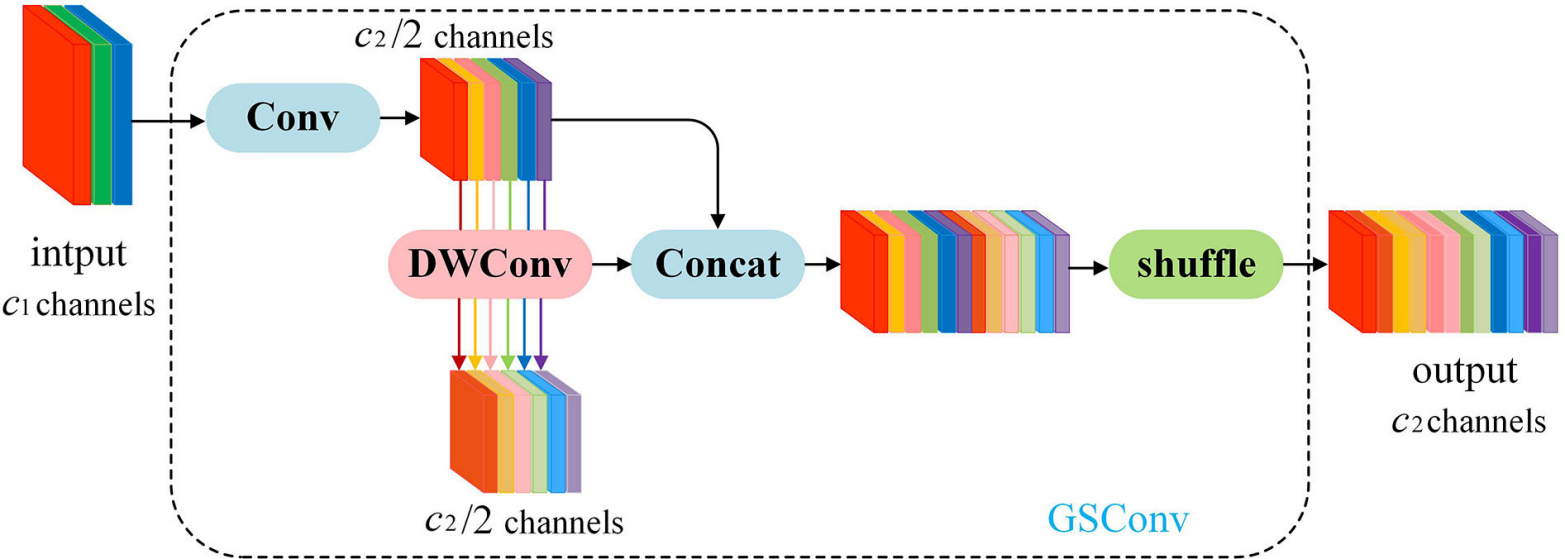
The structure of the GSConv module.

On edge devices, achieving real-time lightweight detection with large models poses challenges. Traditional Depthwise Separable Convolution (DSC) models struggle to achieve high accuracy due to the separation of channel information during computation. This separation diminishes the feature extraction and fusion capabilities of DSC, hindering lightweight high-precision detection. Therefore, GSConv is proposed, merging standard convolution with Depthwise Separable Convolution. The principle involves downsampling with a regular convolution, followed by DWConv depthwise convolution to fuse the results of SCconv and DSCconv, and finally introducing shuffle operations to combine corresponding channels. The structure is illustrated in **Figure 8**.

GSConv has a noticeable impact on lightweight models. Given that the Neck receives feature maps with maximal channel capacity and minimal spatial dimensions, this paper employs GSConv within the Neck. With reduced redundant information in the feature map at this stage, compression is unnecessary, allowing the attention module to operate more effectively, leading to a reduction in model layers and inference time.

**Figure 9:**
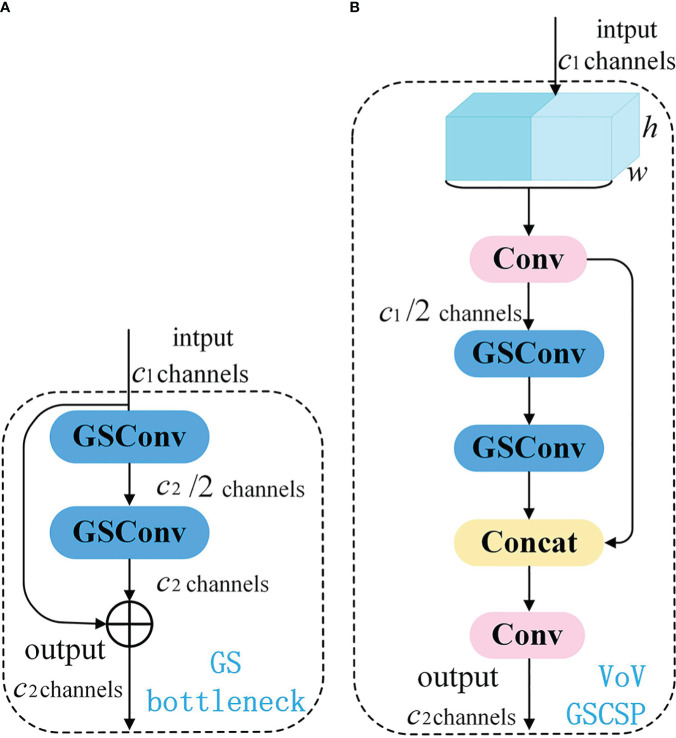
Schematic diagram of Slim-neck paradigm design structure. **(A)** The structures of the GS bottleneck module; **(B)** The VoV-GSCSP modules.

**Figure 10:**
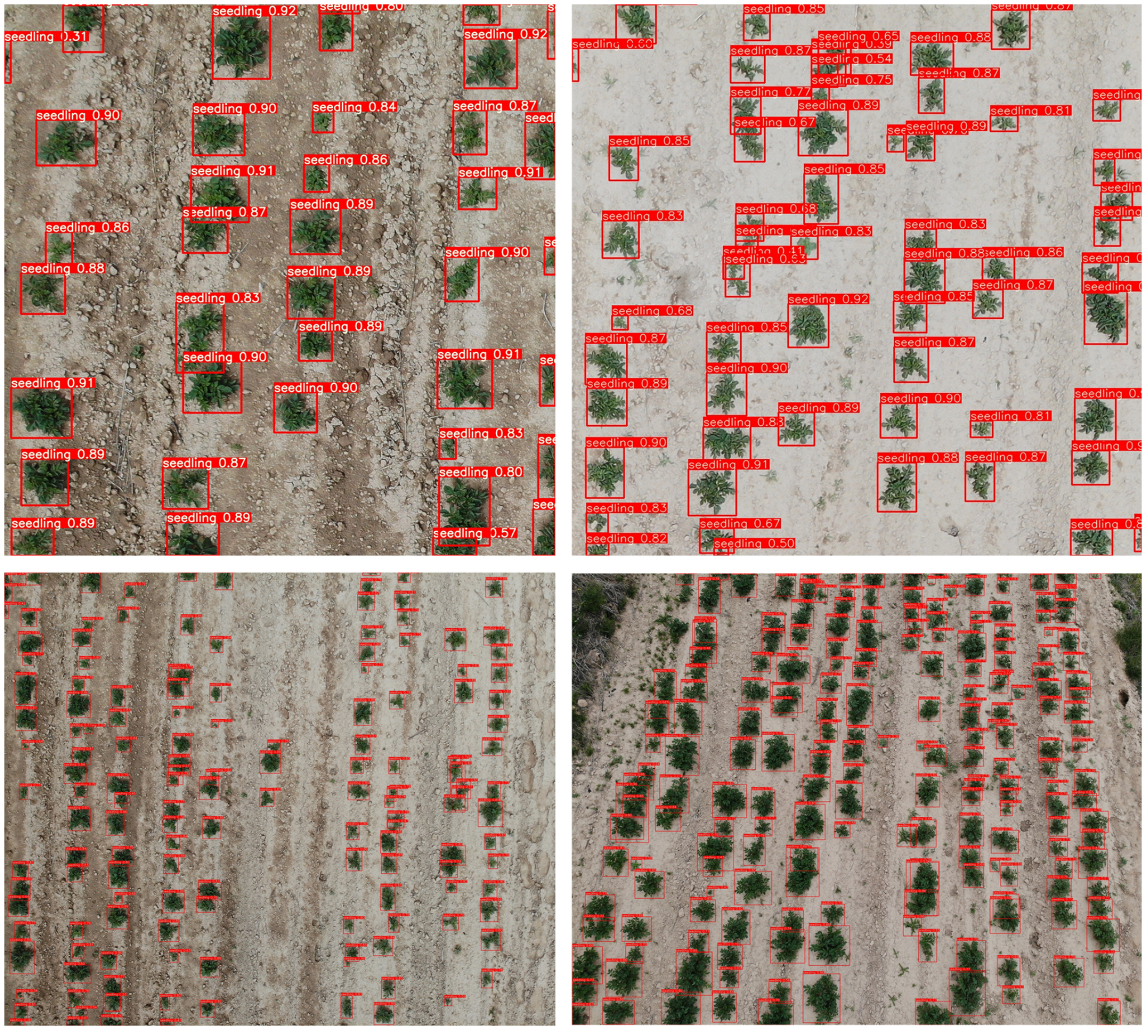
Effect of the detection results after the model is introduced into the BiFPN+GSConv+Slim-neck module.

Introducing GSConv provides a Slim-Neck design paradigm. Initially, this design replaces SC with the lightweight convolution method GSConv in the Neck. GSConv aims to closely match the convolutional computing capability of SC while reducing computational costs. Subsequently, GSbottleNeck is introduced based on GSConv. Similarly, a one-time aggregation method is utilized to design the cross-level subnetwork (GSCSP) module VoV-GSCSP, which simplifies computational and network structural complexity, enhancing detection accuracy. The structure is depicted in **Figure 9**. This paper replaces the C2f module in the YOLOv8 structure with the VoV-GSCSP module to enhance detection performance. After integrating the BIFPN+GSConv+Slim-Neck module, the detection results are illustrated in **Figure 10**.

The detection results demonstrate that the model incorporating BIFPN and GSConv+Slim-Neck achieves high confidence scores when detecting images of seedlings in different environments and growth stages. Nearly all seedling targets are successfully identified, highlighting the feasibility and effectiveness of this improvement method.

### 2.5 Model training and evaluation metrics

#### 2.5.1 Experimental environment

The configuration of the experimental environment and the settings of relevant parameters during the trial process are presented in **Table 1**.

**Table 1:** Experimental environment and related parameter settings.

Training Environment	Details
Programming	Python3.9
Deep learning framework	Pytorch 2.0
GPU	NVIDIA GeForce RTX3060
Operating system	Windows11
img size	640 x 640

#### 2.5.2 Evaluation metrics

This study employs Precision ( P ) in Equation 9, Recall ( R ) in Equation 10, Mean Average Precision ( mAP ) as model accuracy evaluation metrics as in Equation 11, and uses parameters, computation, (i.e., the number of floating-point operations), and Detection Time to measure model complexity and speed. The calculation formulas are as follows.


(9) $$P=\;\frac{TP}{TP+FP}\times 100\%$$

(10) $$R\;=\;\frac{TP}{TP+FN}\times 100\%$$

(11) $$\text{mAP }=\;\frac{\sum_{i=1\;}^{N}\int_{0}^{1}P\left(R\right)\text{d}R}{N}\times 100\%$$

TP represents the number of correctly detected potato sprouts in the image; *TN* represents the number of instances where the model predicts a negative class and the actual label is also negative. FP stands for the count of false detections as potato sprouts; FN indicates the number of missed targets; AP is the Average Precision, represented by the area enclosed by the P-R ( ε=0.0001 ) curve and the coordinate axis; N denotes the number of categories. In this study, only potato sprouts are detected, hence N=1 .

## 3 Results and analysis

### 3.1 VBGS-YOLOv8n ablation experiment

**Figure 11:**
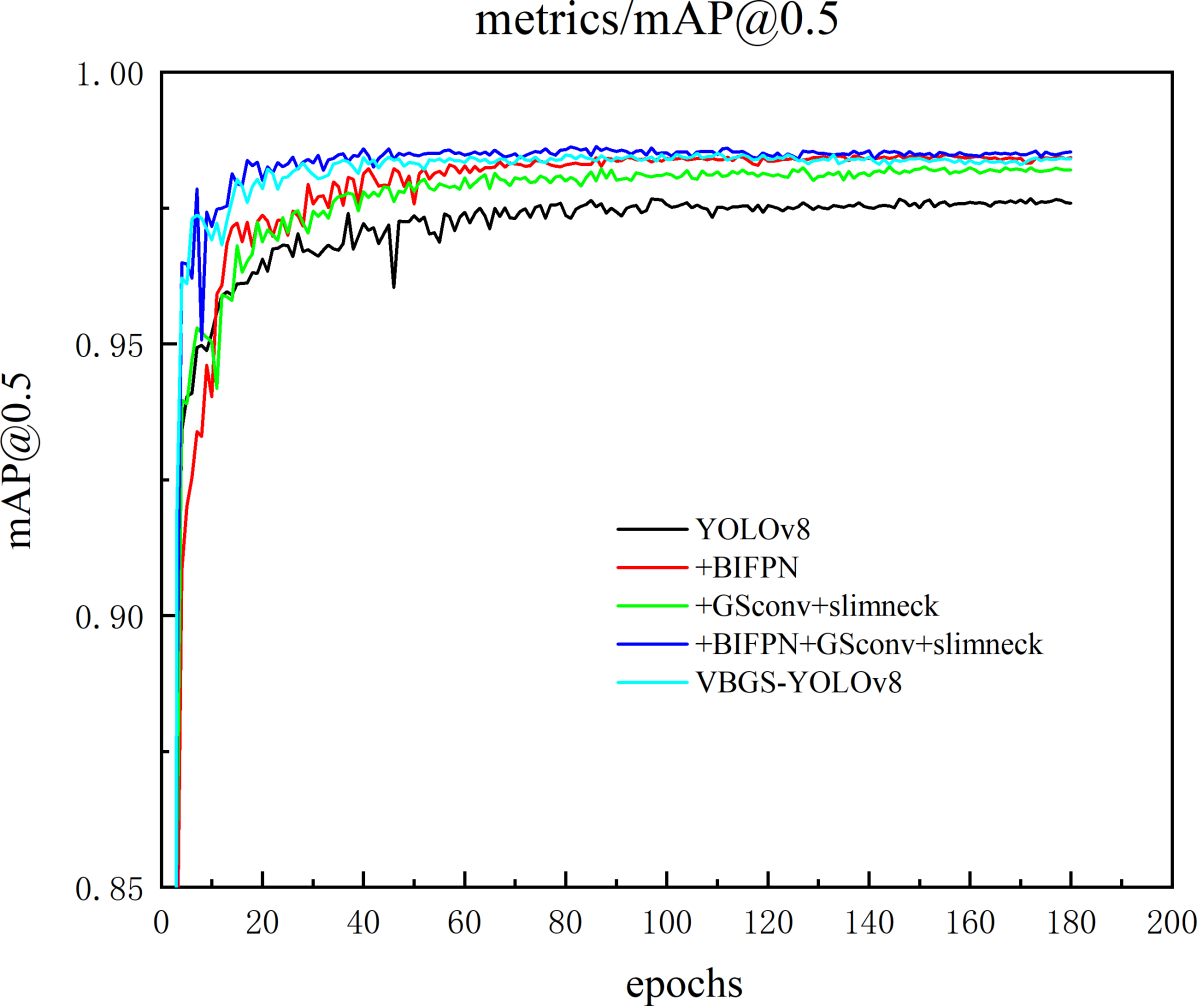
Curve of the model improvement training process.

The VBGS-YOLOv8n model proposed in this study adopts a three-step improvement strategy. Firstly, the BiFPN bidirectional feature pyramid network replaces the PANet pathway aggregation network to enhance feature fusion capabilities and improve small object detection accuracy. Secondly, the GSConv+Slim-Neck is integrated into the Neck section to further enhance model performance. Lastly, to achieve model lightweighting, the main network in the Backbone layer is replaced with the VanillaNet network. To validate the effectiveness of the VBGS-YOLOv8n model in potato seedling detection, this study conducted 7 sets of ablation experiments, with results shown in **Table 2**. Additionally, the training process curve of the model is illustrated in **Figure 11**.

**Table 2:** Comparison of ablation experiment performance.

Model	BiFPN	Gsconv+ slimNeck	VanillaNet	Precision (%)	Recall (%)	mAP (%)		Parameters (M)	Complexity (GFLOPs)	Inference time (ms)
baseline				95.7	96.8	97.6		3157200	8.9	2.9
A	√			96.8	97.3	98.4		3157212	8.9	3.0
B		√		97.1	96.8	98.2		2801619	7.4	2.5
C	√	√		97.0	97.8	98.5		2801631	7.4	2.7
D			√	95.9	96.5	97.5		1644579	5.0	2.3
E	√		√	96.4	96.7	98.0		1644591	5.0	2.4
VBGS-YOLOv8n	√	√	√	97.1	96.2	98.4		1524943	4.2	2.0

From the data in **Table 2**, it is evident that introducing the BiFPN module alone in the original model improves the model’s detection accuracy, recall rate, and mAP value by 1.1, 0.5, and 0.8 percentage points, respectively, albeit with a slight increase in model parameters. When adopting the Gsconv+SlimNeck design paradigm alone, compared to the original YOLOv8n, the model with this module shows an increase of 1.4 and 0.6 percentage points in accuracy and mAP value, respectively. Additionally, the model’s parameter count decreases by 11.3%, computational load significantly reduces, and inference speed improves by 13.8%, indicating a notable enhancement in detection accuracy and model performance. Furthermore, replacing the Backbone network of the original YOLOv8n model with the lightweight VanillaNet network substantially reduces model parameters and computational load, with a 0.2 percentage point increase in accuracy. However, this change leads to a decrease of 0.3 and 0.1 percentage points in recall rate and mAP, respectively. This is attributed to VanillaNet’s lightweight design, which greatly reduces the number of convolutional layer channels and network depth, resulting in decreased performance when handling complex scenes or small targets, thereby impacting recall rate and mean average precision in object detection.

By integrating three improvement strategies, the final outcome of this study is the VBGS-YOLOv8n model. Compared to the original YOLOv8n model, the VBGS-YOLOv8n model shows improvements of 1.4 and 0.8 percentage points in accuracy and mAP, respectively. Additionally, it significantly reduces model parameters and computational load while enhancing inference speed. Specifically, the parameter count is only 48.3% of the original model, the computational load is 47.2% of the original model, and the inference speed increases by 45.0%. However, due to the adoption of the lightweight VanillaNet network, the model’s recall rate decreases by 0.6 percentage points. Nevertheless, considering the study’s focus on potato seedling monitoring, the slight decrease in recall rate, alongside the improved mAP and reduced model complexity, can be deemed negligible in terms of overall effectiveness.

### 3.2 Comparison of detection before and after improvement

**Figure 12:**
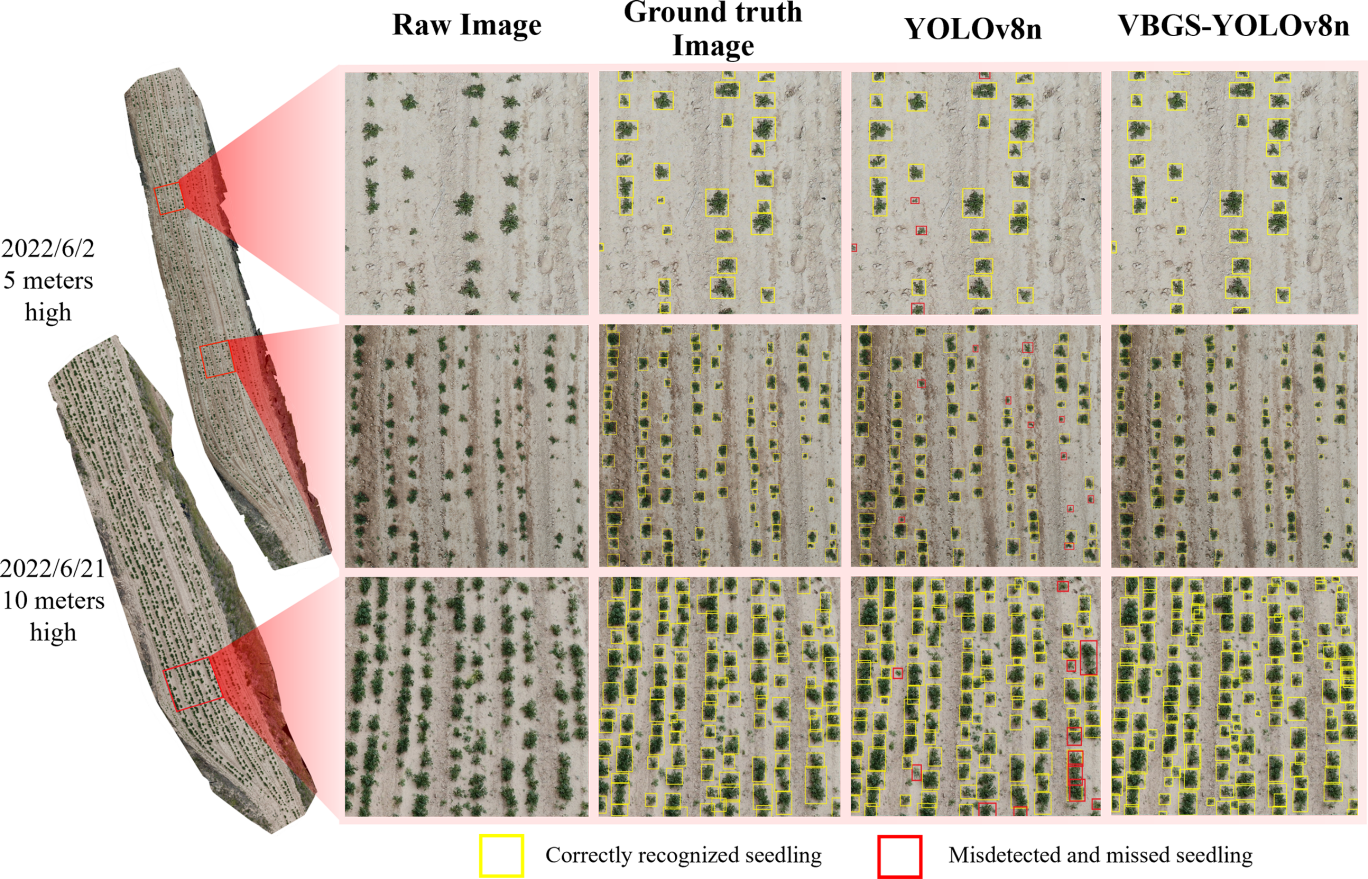
Comparison effect of the model before and after the improvement on the detection of potato seedlings at different heights and at different stages.

The original YOLOv8n network and the improved VBGS-YOLOv8n model were compared on a test set of 220 images. One image of potato seedlings was randomly selected from three different scenarios with varying heights and environmental conditions for demonstration of the detection performance, as shown in **Figure 12**.

The detection results demonstrate the superiority of the VBGS-YOLOv8n model in recognizing various sizes and shapes of potato seedlings, surpassing the original YOLOv8n model significantly. The VBGS-YOLOv8n model can almost entirely identify targets, successfully avoiding instances of missed detections and even detecting overlapping potato seedlings independently. In contrast, the original YOLOv8n model exhibits noticeable issues with missed detections, particularly for smaller potato seedlings in multi-target scenarios, and performs poorly in identifying overlapping potato seedlings.

### 3.3 Comparative horizontal experiment

To further explore the superiority of the VBGS-YOLOv8n network in potato seedling detection, experimental comparisons were conducted between the VBGS-YOLOv8n model and mainstream object detection Network algorithms such as RetinaNet, QueryDet, YOLOv5 and YOLOv8n, as shown in **Table 3**.

**Table 3:** Comparison of experimental results of different network models.

Model	mAP (%)	Parameters (×*10^6^* M)	Complexity (GFLOPs)	FPS
RetinaNet	82.1	28.27	236.28	29.8
QueryDet	90.3	6.61	7.74	37.4
YOLOv5s	95.8	7.20	16.80	68.3
YOLOv7-tiny	94.3	8.90	13.1	51.5
YOLOv8n	97.9	3.16	8.7	90.1
VBGS-YOLOv8n	98.4	1.52	4.2	98.4

**Figure 13:**
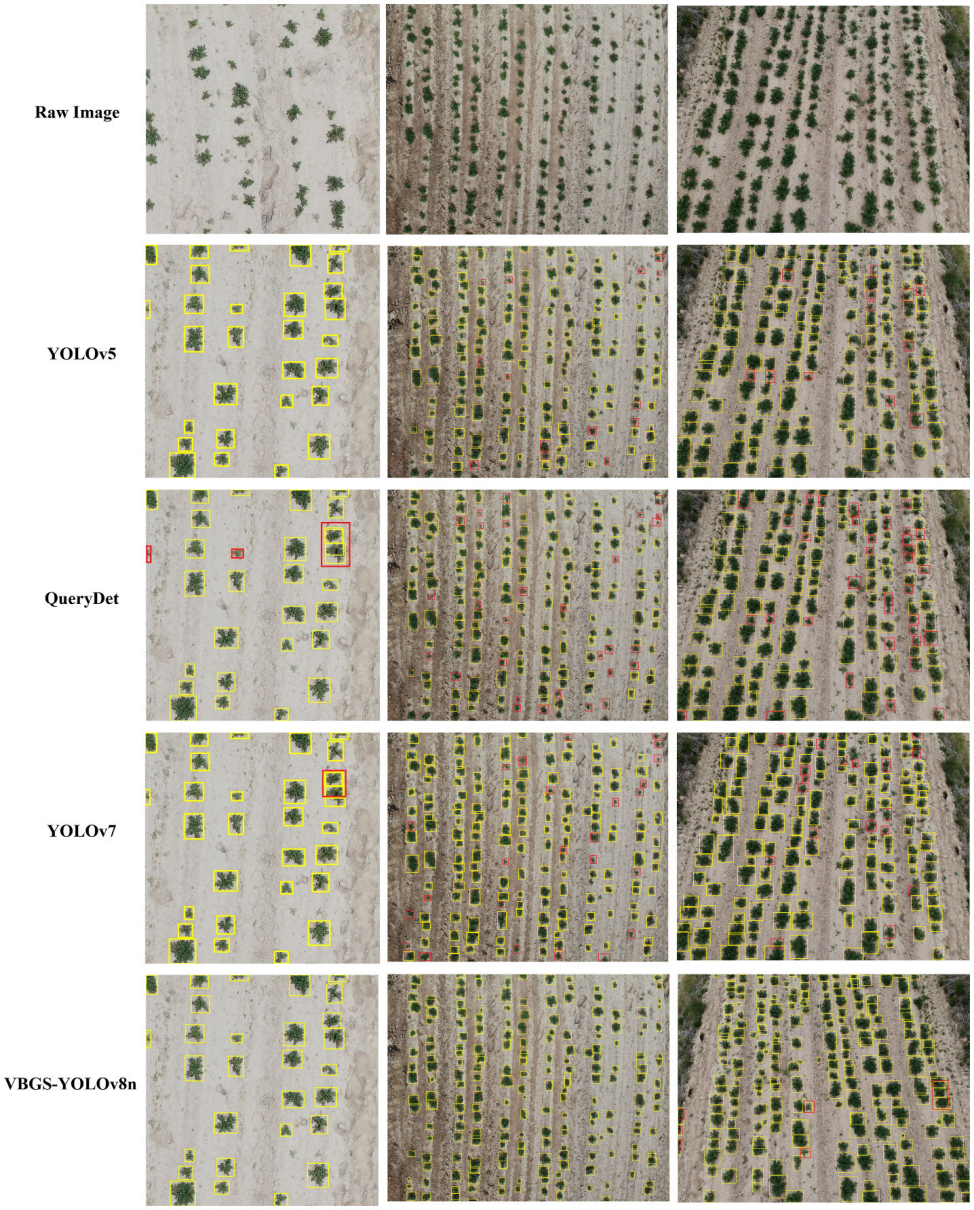
Detection results of potato seedlings in different environments by different models.

From the table data, it is evident that compared to mainstream models, the VBGS-YOLOv8n network surpasses current mainstream detection models in all performance metrics, with a significant improvement in mAP. More importantly, while maintaining high performance, the VBGS-YOLOv8n model has the lowest parameter count and computational load, further highlighting its superiority and efficiency. RetinaNet, despite using FPN and a new focal loss function to enhance model efficiency and run on low-end devices, faces accuracy issues in small object detection and has high computational load, making it unsuitable for this experiment. QueryDet, a small object detection model that accelerates feature pyramid object detector inference speed using a novel query mechanism, employs the Sparse Cascaded Query (CSQ) mechanism to obtain high-resolution feature maps while minimizing computation on background regions. Comparing QueryDet to RetinaNet in the table data, QueryDet shows improvements in all metrics, with optimal parameter and computational load compared to other mainstream models, with computational load only 3.54 points higher than the VBGS-YOLOv8n model in this study. However, its detection accuracy is 8.3 percentage points lower than the model in this study. YOLOv5, another model in the YOLO series widely used for its good performance and detection results, shows comparable detection accuracy to the method in this study but with increased complexity and lower inference speed, making it unsuitable for mobile deployment and potato seedling detection. YOLOv7-tiny, the latest algorithm in the YOLO series, achieves decent accuracy with fewer parameters and computational load, but its FPS is 48% lower than the proposed new method, indicating slower model detection speed. The experimental data comparison underscores the superiority and efficiency of the VBGS-YOLOv8n network, which not only meets the accuracy requirements but also features a more lightweight network architecture suitable for potato seedling detection scenarios. The comparative detection performance of different models is illustrated in **Figure 13**.

The results indicate that the improved lightweight model outperforms other object detection models in recognizing potato seedlings at different growth stages and heights. It accurately locates potato seedlings, which are dense small targets. In the images, the detection labels and confidence scores were removed for clarity, but in the experiment, detections exhibited high confidence. The predicted bounding boxes fully encapsulate the potato seedlings, even identifying overlapping instances without any missed detections. In the case of the first set of photos with fewer targets at a height of 5 meters, where the potato seedlings are larger and less dense, both YOLOv5 and YOLOv7 in the YOLO series can detect all targets effectively. However, YOLOv7 shows some instances of redundant bounding boxes, indicating slightly inferior detection performance compared to YOLOv5. For small targets at the corners, QueryDet exhibits some missed detections. In the detection results for the other two environments, it is evident that the proposed VBGS-YOLOv8n model has the fewest missed detections and minimal redundant bounding boxes. This clearly demonstrates the excellent performance and accuracy of the VBGS-YOLOv8n model in recognizing potato seedlings.

## 4 Conclusion

This study introduces an enhanced VBGS-YOLOv8n network, aimed at addressing the challenge of detecting potato seedlings in drone remote sensing imagery. The model utilizes the lightweight VanillaNet algorithm as its backbone, effectively reducing the model’s complexity. It incorporates a BiFPN to improve the retention of detailed features, thereby enhancing the accuracy of small target detection. GSconv convolution is employed in the neck to maintain overall accuracy, and the VoV-GSCSP network replaces all C2f modules in the original YOLOv8n algorithm’s neck, significantly reducing the model’s parameter count. Experimental validation demonstrates that VBGS-YOLOv8n exhibits exceptional performance in detecting small targets, with accuracy and mAP reaching 97.1% and 98.4%, respectively. Compared to the original YOLOv8 model, there is a 1.4% increase in accuracy and a 0.8% increase in mAP, alongside a 31.0% reduction in computation time. The parameter count is 48.3% of the original model, and the computational load is only 47.2%, with significant reductions in both missed and false detections. To verify its effectiveness, comparative analyses with leading models in the field affirm its superior detection accuracy, efficiency in parameter usage, and overall performance. The VBGS-YOLOv8n model achieves an optimal balance between detection speed, accuracy, and size, rendering it ideal for deployment on agricultural mobile devices. Future work will focus on optimizing the model for practical drone applications and broader datasets, ensuring the feasibility of VBGS-YOLOv8n and its detection capabilities for similar small target crops, offering technical support for precision agriculture.

## Data availability statement

The original contributions presented in the study are included in the article/supplementary material. Further inquiries can be directed to the corresponding author.

## Author contributions

LW: Methodology, Resources, Software, Writing – review & editing. GW: Conceptualization, Formal analysis, Funding acquisition, Methodology, Validation, Writing – original draft, Writing – review & editing. SY: Conceptualization, Funding acquisition, Software, Writing – review & editing. YL: Data curation, Resources, Writing – original draft, Writing – review & editing. XY: Writing – review & editing. BF: Funding acquisition, Visualization, Writing – review & editing. WS: Conceptualization, Formal analysis, Writing – review & editing. HL: Investigation, Validation, Writing – review & editing.
